# The SECI model of knowledge creation as an enabler of students’ creative behavior through the lens of absorptive capacity

**DOI:** 10.3389/fpsyg.2025.1708055

**Published:** 2026-01-09

**Authors:** Lifen Han, Yanwu Zhao

**Affiliations:** 1 Department of Education and Psychological Sciences, Yuncheng University, Yuncheng, China; 2 School of Education, Handan University, Handan, China

**Keywords:** SECI model, absorptive capacity, acquisition, assimilation, transformation, student creativity, higher education, PLS-SEM

## Abstract

**Introduction:**

The present study investigates how the SECI model of knowledge creation enables students’ creative behavior through absorptive capacity pathways.

**Methods:**

Drawing on data from 395 university students in China collected across three waves, the research applies partial least squares structural equation modeling (PLS-SEM) to examine both lower-order and higher-order representations of SECI.

**Results:**

The results show that SECI significantly enhances acquisition, assimilation, and transformation, confirming its predictive role in absorptive capacity development. In addition, mediation analysis reveals that acquisition and transformation serve as significant pathways linking SECI to students’ creative behavior, while assimilation, although positively predicted by SECI, does not directly foster creativity.

**Discussion:**

These findings contribute to absorptive capacity theory by clarifying the relative strength of its pathways and extend the SECI framework by validating it as a higher-order construct in educational contexts. Beyond theoretical contributions, the study highlights practical implications for higher education institutions: fostering environments that emphasize knowledge acquisition and transformation, alongside integrated SECI processes, can enhance students’ creative competencies.

## Introduction

Creativity has increasingly been recognized as a cornerstone of success in contemporary education, shaping how students adapt to rapid technological change and complex social challenges (Fischer-Schöneborn et al., 2025). In knowledge-driven environments, universities are under mounting pressure to cultivate not only disciplinary knowledge but also the capacity for novel problem-solving, adaptability, and innovation (Brauer et al., 2024). Scholars argue that creativity is not an isolated trait but the outcome of dynamic knowledge processes that enable individuals to generate, refine, and apply new ideas (Fan and Cai, 2022; Gilson, 2024; Wan et al., 2021). This recognition has sparked growing interest in frameworks explaining how individuals create, share, and utilize knowledge to support creative outcomes. Yet, much of this discourse remains fragmented, as prior studies often examine knowledge mechanisms in isolation rather than as an integrated system influencing creativity.

One of the most influential frameworks for understanding knowledge creation is the SECI model proposed by Nonaka et al. (1996). The model explains how tacit and explicit knowledge are continuously converted through socialization, externalization, combination, and internalization, forming a spiraling process of knowledge evolution (Bandera et al., 2017). SECI has been widely applied in organizational research to explain innovation, but its potential in higher education remains underexplored (Barreto and Abarca, 2025; Żatuchin, 2025). Although emerging studies highlight the value of SECI processes in digital learning and collaborative contexts, the literature remains theoretically inconsistent, with most studies treating SECI as four separate activities rather than a unified capability. This limits clarity on how SECI functions holistically to shape student creativity.

Absorptive capacity (ACAP), defined as the ability to acquire, assimilate, transform, and exploit knowledge (Zahra and George, 2002), offers a complementary perspective for understanding how knowledge contributes to creative performance. Recent work suggests that ACAP helps explain how learners process and integrate knowledge in educational settings (Lenart-Gansiniec et al., 2022; Lo and Tian, 2020). However, previous findings remain mixed, especially regarding whether different ACAP pathways contribute equally to creative outcomes. Some studies indicate stronger effects for acquisition and transformation, while others emphasize comprehension-based pathways, revealing conceptual ambiguity. This raises critical questions about how different absorptive mechanisms operate within SECI-driven learning environments.

To further strengthen the theoretical grounding, this study also draws on the Knowledge-Based View (KBV), which positions knowledge as the most strategic resource driving innovation, capability development, and performance. KBV argues that organizations—and by extension, learners—derive advantage from their ability to create, integrate, and apply knowledge (Grant, 1996; Spender, 1996). Integrating KBV with SECI and ACAP provides a coherent lens to explain why dynamic knowledge processes should predict creative behavior, particularly in higher education where knowledge acts as both input and output of learning.

Despite progress in understanding these frameworks individually, several gaps remain. First, prior research rarely integrates SECI and ACAP into a single explanatory model, leaving unclear how knowledge creation interacts with absorptive mechanisms to produce creativity. Second, evidence in higher education is limited, with most findings drawn from organizational or qualitative studies. Third, the role of unequal ACAP pathway contributions remains theoretically unresolved. Finally, few studies validate SECI as a higher-order construct using robust, time-lagged quantitative designs. These gaps restrict a comprehensive understanding of how knowledge processes shape students’ creative behavior.

To address these gaps, this study investigates how SECI processes influence student creativity through acquisition, assimilation, and transformation pathways. Based on the above gaps, the following research questions guide this investigation:

- *RQ1: How does the SECI model of knowledge creation influence students’ creative behavior?*
- *RQ2: Do absorptive capacity pathways mediate the relationship between SECI and creative behavior?*
- *RQ3: Which absorptive pathways (acquisition, assimilation, transformation) exert the strongest influence on creativity?*
- *RQ4: Does conceptualizing SECI as a higher-order construct improve its explanatory power in predicting creative behavior*?

By integrating SECI, ACAP, and KBV into a unified framework, this study provides a sharper theoretical foundation and offers a more coherent explanation of how dynamic knowledge processes contribute to creativity in higher education.

## Literature

### Theoretical underpinnings

#### Knowledge creation

Knowledge creation and utilization have long been recognized as pivotal processes for fostering innovation and creativity in organizations and education alike (Nonaka and Toyama, 2003). Among the diverse theories addressing how knowledge evolves, the SECI model remains foundational, positing that socialization, externalization, combination, and internalization form a continuous spiral of knowledge conversion between tacit and explicit forms (Bandera et al., 2017). This framework highlights not only the importance of interpersonal interactions in sharing tacit knowledge but also the role of structured articulation and reconfiguration of ideas in shaping innovative outcomes (Żatuchin, 2024). Within educational contexts, these processes offer explanatory power for how students develop, exchange, and internalize insights that may later manifest as creative behavior (Fischer-Schöneborn et al., 2025).

However, despite its widespread adoption, SECI research in education remains theoretically fragmented and often overly descriptive. Studies frequently focus on isolated SECI components rather than the dynamic interplay of the entire cycle, which limits understanding of how knowledge conversion processes collectively influence learning outcomes (Żatuchin, 2024). Moreover, empirical findings are inconsistent: while some studies demonstrate strong links between SECI processes and creativity (Asbari and Asbari, 2025), others suggest that these effects vary across cultural, pedagogical, and technological contexts (Maras et al., 2024). This inconsistency raises questions about whether SECI operates uniformly across learning environments or whether its effects depend on contextual moderators. Additionally, much of the existing work relies on qualitative case studies or descriptive analyses, creating a methodological gap concerning the model’s higher-order validity in student populations (Guzik et al., 2025). These limitations highlight the need for more rigorous and integrated assessments of SECI in educational research.

#### Absorptive capacity

Absorptive capacity provides a complementary theoretical foundation. Originally conceptualized as a firm’s ability to recognize, assimilate, and apply external knowledge (Camisón and Forés, 2010), it was later reconceptualized into four dimensions: acquisition, assimilation, transformation, and exploitation (Zahra and George, 2002). This reconceptualization distinguishes between potential absorptive capacity (acquisition and assimilation) and realized absorptive capacity (transformation and exploitation), emphasizing that innovation requires not just learning but also reconfiguring and applying knowledge in practice (Balle et al., 2020). In recent years, absorptive capacity has gained traction in educational research, where it explains how learners engage with knowledge sources, process them meaningfully, and eventually generate novel outputs (Lenart-Gansiniec et al., 2022; Lo and Tian, 2020).

From a KBV perspective, absorptive capacity represents a core capability that enables actors to convert knowledge resources into meaningful performance outcomes. KBV posits that competitive and innovative advantages emerge when individuals or organizations develop the ability to integrate, interpret, and deploy knowledge effectively (Grant, 1996; Spender, 1996). In this sense, absorptive capacity acts as the mechanism through which knowledge is transformed into action, making it a critical capability for creativity in educational contexts.

Furthermore, recent empirical studies reinforce the strategic role of absorptive capacity in driving innovative outcomes. Hassan et al. (2025) demonstrate that knowledge absorptive capacity plays a pivotal mediating role between high-performance work systems and innovation performance, highlighting that ACAP is not merely a cognitive resource but a value-creation capability. Similarly, Hassan et al. (2023) show that absorptive and intellectual capabilities jointly enable ambidexterity and innovation, offering strong evidence that learning pathways operate sequentially and interactively—an insight directly aligned with this study’s serial mediation logic. Earlier conceptual work by Hassan et al. (2017) also argues that knowledge-driven operational capabilities mediate the relationship between intellectual capital and performance, reinforcing the idea that ACAP converts knowledge inputs into meaningful outcomes.

In the educational domain, these insights underscore that students’ absorptive capacity is not merely about receiving information but about developing the capability to reinterpret, transform, and apply knowledge creatively. This is consistent with Abrar et al. (2021), who found that knowledge-based training interventions substantially promote innovative behavior, suggesting that learners with higher absorptive abilities are more capable of turning learning experiences into novel outputs.

Despite its relevance, existing studies often treat ACAP components descriptively, without critically assessing whether all four pathways contribute equally to creativity. Evidence remains inconsistent: some studies highlight acquisition and transformation as strong predictors of innovation, whereas assimilation is frequently interpreted as a background cognitive process with weaker behavioral implications. These inconsistencies indicate unresolved theoretical tensions regarding the relative weight of each ACAP dimension—making it essential to test these pathways concurrently within an integrated framework.

#### Creativity

Creativity theories further enrich our theoretical framework. In our study, creativity has been framed as the outcome of interactions between knowledge inputs, cognitive processing, and contextual enablers (Tang et al., 2022). The componential theory of creativity, for example, stresses that domain-relevant skills and knowledge underpin creative performance, but that these must be flexibly applied and recombined in response to new problems (Fan and Cai, 2022). Absorptive capacity (Zahra and George, 2002) thus provides the mechanism, while SECI (Nonaka and Toyama, 2003) provides the context in which students generate and refine the knowledge required for creativity. Together, these frameworks suggest that dynamic knowledge processes, when activated in educational environments, should enable creative behaviors that prepare students for complex societal and professional challenges (Fischer-Schöneborn et al., 2025).

From a KBV lens, creativity emerges not only from knowledge possession but from the capability to mobilize, recombine, and exploit knowledge for novel purposes (Grant, 1996; Spender, 1996). This aligns with the argument that creativity represents a knowledge-application outcome rather than a standalone psychological trait. In other words, learners become creative when they harness and transform knowledge resources through structured processes such as SECI and capability-based mechanisms such as absorptive capacity.

Empirical work provides additional support for this view. Abrar et al. (2021) found that knowledge-based training and development significantly enhance innovative behavior, reinforcing the premise that creativity is a knowledge-driven outcome. Similarly, Hassan et al. (2023) showed that intellectual capital and ambidextrous capabilities jointly stimulate innovation performance, implying that creativity and innovation stem from integrated knowledge pathways rather than isolated abilities. These insights suggest that creativity in education likely arises from the interaction between knowledge creation processes (SECI) and knowledge processing capabilities (ACAP).

Despite these advancements, the creativity literature in higher education often remains descriptive rather than explanatory, with limited focus on how structured knowledge mechanisms translate into creative outputs. Many studies emphasize individual traits or environmental stimuli but give insufficient attention to how knowledge flows, conversions, and absorptive mechanisms shape creativity. This indicates a theoretical gap, particularly in understanding the sequential and mediating processes through which knowledge is reconstructed and applied to produce novel student behaviors. Addressing this gap requires integrative models that combine SECI, absorptive capacity, and creativity within a unified theoretical logic—a direction this study aims to advance.

#### SECI model of knowledge creation and creative behavior

Creativity is widely recognized as the capacity to generate ideas that are both novel and useful, and it is increasingly seen as essential for students navigating complex, knowledge-intensive environments (Brauer et al., 2024). Knowledge creation processes are at the heart of this capability, as they shape how learners develop, refine, and apply their ideas in innovative ways (Bandera et al., 2017; Cerchione et al., 2024). Among these, the SECI model of knowledge creation (Nonaka et al., 1996) provides a powerful framework for understanding how creativity emerges from the continuous conversion of tacit and explicit knowledge. Through socialization, individuals share experiences and tacit insights; externalization enables articulation of hidden knowledge into explicit forms; combination integrates multiple knowledge sources; and internalization embeds knowledge through reflection and practice (Guzik et al., 2025). Together, these processes create fertile ground for creativity, as they not only expand knowledge resources but also stimulate their dynamic recombination.

Despite its theoretical promise, existing evidence on SECI’s effectiveness in enhancing student creativity is not fully consistent. Some studies demonstrate strong positive effects (Asbari and Asbari, 2025; Żatuchin, 2025), yet others report more modest or context-dependent relationships, suggesting that SECI may not uniformly translate into creativity across learning environments (Maras et al., 2024; Meza et al., 2024). Methodological differences also contribute to mixed findings, as many studies examine individual SECI dimensions in isolation rather than evaluating the full integrated cycle. This fragmented empirical base underscores the need for models that assess SECI holistically and test its predictive validity using robust analytical approaches.

Empirical research has begun to highlight how SECI processes enhance creative outcomes in education. Asbari and Asbari (2025) shows that embedding SECI principles into digital course design helps students transform tacit experiences into new conceptual understandings, which later translate into creative project work. Similarly, Żatuchin (2025) emphasize that absorptive and knowledge-creation capacities in schools provide the foundation for creative learning, as students who actively share, articulate, and integrate knowledge display higher innovation in problem-solving tasks. These findings resonate with organizational studies demonstrating that SECI activities directly support innovative performance by fostering conditions of openness, dialogue, and knowledge recombination (Maras et al., 2024; Meza et al., 2024).

Theoretically, SECI creates the preconditions for creativity by enabling knowledge diversity and dynamic interaction (Gilson, 2024). Socialization exposes learners to new perspectives that fuel idea generation, while externalization requires them to clarify and refine their thoughts in communicable form. Combination broadens cognitive repertoires by merging diverse information sets, and internalization ensures that new insights become embodied in routines and personal skills. Each process not only enhances knowledge but also prompts experimentation, reflection, and recombination—all central to creative behavior (Wan et al., 2021). Building on these perspectives, it is reasonable to expect that students engaged in SECI processes will be more likely to demonstrate creative behavior. However, scholars also note that SECI’s impact may manifest indirectly through deeper cognitive processes rather than producing immediate creative outputs (Wan et al., 2021; Cerchione et al., 2024). This suggests that SECI provides the knowledge context, but additional mechanisms—such as the absorptive capacity pathways—may be required to activate creativity fully. Thus,

*H1*: The SECI model of knowledge creation positively influences creative behavior.

#### Linking SECI processes with acquisition

The SECI model emphasizes that knowledge creation begins with processes of interaction and conversion that expose individuals to new ideas and experiences (Nonaka et al., 1996). Acquisition, as the first dimension of absorptive capacity, reflects an individual’s ability to identify and obtain external knowledge deemed valuable for learning and performance (Zhang et al., 2025). When SECI processes are activated—particularly socialization and externalization—they enhance opportunities for students to engage with diverse knowledge sources, thereby strengthening acquisition capabilities (Żatuchin, 2024). For example, collaborative sharing of tacit insights in peer groups and the articulation of ideas into explicit forms create entry points for capturing new knowledge inputs that would otherwise remain inaccessible (Lee, 2022).

Empirical research provides evidence that structured knowledge creation activities enrich acquisition. Recent studies show that digital learning environments that integrate SECI-inspired processes lead students to actively seek and internalize new knowledge resources (Cerchione et al., 2024). Similarly, Fischer-Schöneborn et al. (2025) demonstrate that absorptive capacity in educational contexts, particularly acquisition, is enhanced when schools build collaborative cultures of sharing and openness to external ideas. Within higher education, acquisition is further stimulated by SECI’s combination process, where knowledge from different sources is integrated, prompting learners to actively search for and capture complementary inputs (Guzik et al., 2025). This aligns with the premise that knowledge creation processes serve as enablers of absorptive capacity, ensuring that acquisition is not a passive act but a deliberate pursuit of relevant knowledge.

Theoretical insights further reinforce this link. For instance, absorptive capacity theory highlights that acquisition requires prior related knowledge, motivation, and exposure to diverse information channels (Lenart-Gansiniec et al., 2022; Lo and Tian, 2020). SECI processes provide the mechanisms through which these requirements are fulfilled: socialization builds the experiential basis, externalization fosters articulation and codification, combination encourages integration, and internalization embeds the acquired knowledge into individuals’ routines. Thus, SECI is expected to act as a catalyst that triggers and strengthens acquisition among students, equipping them with the resources needed for creativity and innovation.

Yet, some studies caution that acquisition may depend heavily on contextual factors such as learning climate, motivation, and availability of knowledge resources (Lenart-Gansiniec et al., 2022; Lo and Tian, 2020). Without supportive environments, SECI processes may generate interaction opportunities but not necessarily translate into stronger acquisition behaviors. These inconsistencies highlight the importance of examining acquisition within structured SECI-rich settings rather than assuming a universal positive effect. Building on these arguments, it is reasonable to hypothesize that:

*H2*: The SECI model of knowledge creation positively influences acquisition.

#### Linking SECI processes with assimilation

Assimilation represents the ability to analyze, interpret, and internalize acquired knowledge so that it can be effectively understood and integrated into existing cognitive structures (Zahra and George, 2002). It is considered part of potential absorptive capacity, reflecting comprehension rather than application. The SECI model, particularly through combination and internalization, provides a rich context for assimilation to occur (Barreto and Abarca, 2025). When students combine explicit knowledge from diverse sources, they restructure and reinterpret information in meaningful ways. Internalization further embeds this understanding by transforming explicit knowledge into tacit forms through practice and reflection, thereby deepening comprehension (Mohamed, 2021).

Prior studies highlight the relevance of SECI processes in strengthening assimilation. For instance, Avdimiotis et al. (2022) demonstrated that structured knowledge activities increase individuals’ ability to make sense of new information by contextualizing it within their prior knowledge base. Recent educational research also suggests that environments built around SECI principles enhance students’ capability to understand complex ideas, as the iterative cycle of articulation, combination, and reflection facilitates sense-making (Asbari and Asbari, 2025; Żatuchin, 2025). Moreover, Fischer-Schöneborn et al. (2025) argue that absorptive capacity in education is particularly dependent on the interpretive abilities of learners, which are activated when they are exposed to collaborative and knowledge-rich contexts—conditions that SECI explicitly fosters.

From a theoretical standpoint, assimilation requires both cognitive readiness and contextual enablers. In this perspective, Zhang et al. (2025) originally stressed that an individual’s prior knowledge base determines their ability to assimilate new inputs, but this capacity is magnified when structures exist to help decode and internalize information. Seemingly, SECI provides such structures by creating spaces for dialogue, codification, and integration of knowledge into practice (Wan et al., 2021). For example, socialization may trigger questions that require interpretation, while externalization pushes individuals to refine their understanding in language others can grasp. Together, these processes generate a cycle of comprehension that strengthens assimilation. Furthermore, in educational contexts, assimilation is essential for enabling students to progress beyond rote memorization toward meaningful understanding (Cheng, 2023). By supporting interpretation, reflection, and integration of knowledge, SECI is expected to enhance students’ assimilation capability.

However, empirical findings on assimilation remain mixed. Some studies show strong interpretive benefits, while others argue that assimilation alone may not lead to higher-level outcomes unless coupled with transformation or application (Cerchione et al., 2024). This suggests that SECI may strengthen assimilation, but its effectiveness depends on whether comprehension is subsequently used for deeper cognitive restructuring. These tensions highlight the need to study assimilation in relation to other absorptive pathways rather than in isolation. Therefore, building on both theoretical reasoning and empirical evidence, the following hypothesis is inferred:

*H3*: The SECI model of knowledge creation positively influences assimilation.

#### Linking SECI processes with transformation

Transformation refers to the ability to refine, reconfigure, and integrate new knowledge with existing cognitive structures in order to create novel insights (Zahra and George, 2002). It represents a critical stage of realized absorptive capacity, emphasizing not just comprehension but the reshaping of knowledge for future application. The SECI model, particularly through combination and externalization, plays a central role in enabling transformation by fostering conditions where explicit and tacit knowledge are continuously reorganized and adapted to emerging contexts (Li et al., 2018). When students combine knowledge from multiple sources and articulate their perspectives in new forms, they are actively transforming knowledge into resources that can support innovation.

Previous research underpins the importance of transformation as a bridge between knowledge acquisition and creative outcomes. Obeidat (2019) argue that transformation is the mechanism through which organizations adapt prior knowledge to align with new external information, ensuring relevance and novelty. Extending this perspective, Cerchione et al. (2024) demonstrate that transformation involves experimentation and reinterpretation, processes that parallel SECI’s emphasis on recombining and recontextualizing knowledge. More recent studies in educational contexts support this argument. Guzik et al. (2025) finds that digital learning designs grounded in SECI enable students to restructure their knowledge frameworks, while Kastelli et al. (2024) note that absorptive capacity in schools is heightened when learners actively transform knowledge rather than merely assimilate it.

Yet, transformation’s development may vary significantly across learners and contexts. Some studies note that transformation emerges only when learners possess sufficient prior understanding and opportunities for reflection (Wan et al., 2021). Others caution that transformation is resource-intensive and may only occur in highly supportive knowledge environments (Maras et al., 2024). These contrasting findings illustrate the need to examine transformation within well-structured SECI activities rather than assuming a uniform effect.

Given these arguments, SECI is expected to exert a strong positive influence on transformation. By embedding students in cycles of knowledge conversion and recombination, SECI equips them with the ability to restructure ideas in ways that promote novelty and innovation. Therefore, it is reasonable to hypothesize:

*H4*: The SECI model of knowledge creation positively influences transformation.

#### Mediating roles of absorptive capacity pathways

While the SECI model directly stimulates creative behavior, its influence often unfolds through deeper cognitive mechanisms that determine how students capture, interpret, and reconfigure knowledge. Absorptive capacity provides this explanatory lens, clarifying how the movement from knowledge creation to creativity is enabled by pathways of acquisition, assimilation, and transformation (Tallarico et al., 2025). Rather than operating in isolation, these pathways form an interconnected process in which students first access relevant knowledge, then make sense of it, and finally reshape it for novel application. Together, they constitute the channels through which SECI’s dynamic knowledge conversion translates into creativity.

The first step in this chain is acquisition—the capacity to identify and absorb valuable external inputs (Fernández-Mesa et al., 2022). SECI’s socialization and externalization processes cultivate environments of interaction and dialogue that encourage students to seek new knowledge from peers, instructors, and external resources. Such exposure broadens their informational base and equips them with diverse material for idea generation. Indeed, recent research has shown that students embedded in SECI-rich settings actively acquire knowledge that later becomes the raw material for creativity (Asbari and Asbari, 2025). However, acquisition may not always be sufficient for creativity unless students are supported with opportunities to deepen and process what they acquire (Lenart-Gansiniec et al., 2022).

Yet acquisition alone is insufficient without deeper processing. Assimilation ensures that the knowledge acquired through SECI cycles is interpreted, organized, and meaningfully connected to prior understandings (Mardiani and Samsuryadi, 2023). Combination and internalization processes within SECI support this integrative effort, helping students contextualize and embed external information into their cognitive schemas. While comprehension does not always produce immediate creative breakthroughs, it provides the interpretive depth that allows students to recognize patterns and prepare for future novelty (Barreto and Abarca, 2025). Assimilation thus represents a quieter but essential pathway: it mediates the SECI–creativity link by ensuring that knowledge inputs are not merely collected but cognitively anchored.

Transformation then builds on this foundation by reconfiguring both acquired and assimilated knowledge into new forms that can generate originality. SECI’s emphasis on combination and externalization provides the conditions for this restructuring (Żatuchin, 2025), prompting students to articulate insights and merge diverse perspectives into innovative solutions. Transformation has been consistently linked to creativity because it represents the stage at which learners move beyond understanding toward novelty creation (Wang and Zhang, 2024). By encouraging learners to reorganize knowledge and apply it in fresh contexts, SECI indirectly fosters creative behavior through the transformative power of absorptive capacity.

**Figure 1:**
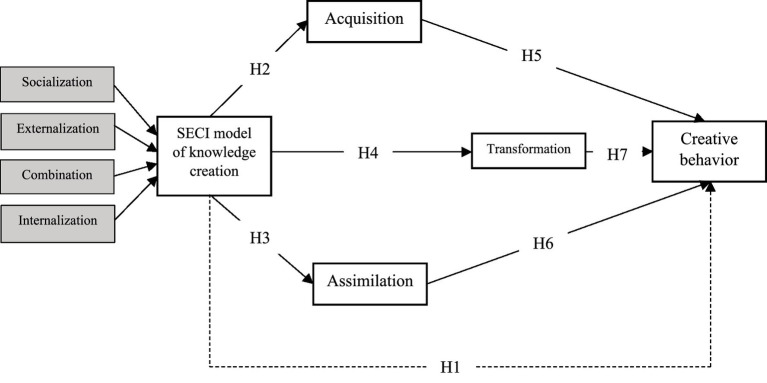
Reflective-reflective hierarchical model.

Taken together, these absorptive pathways illustrate that the relationship between SECI and creativity is not merely direct but is also carried forward through a chain of knowledge processes (Figure 1). Acquisition opens access to new resources, assimilation secures their interpretive grounding, and transformation converts them into innovative outputs. Each plays a distinct role, yet collectively they form the mediating mechanism by which SECI enhances creativity. Therefore, it is proposed that:

*H5*: Acquisition mediates the relationship between the SECI model of knowledge creation and creative behavior.

*H6*: Transformation mediates the relationship between the SECI model of knowledge creation and creative behavior.

*H7*: Assimilation mediates the relationship between the SECI model of knowledge creation and creative behavior.

## Method

The study adopted a quantitative research design using a time-lagged survey approach to minimize common method bias and to capture the dynamic links between knowledge creation, absorptive capacity, and students’ creative behavior (Creswell and Creswell, 2017; Podsakoff et al., 2003). The target population comprised university students in China, a context where higher education increasingly emphasizes collaborative and knowledge-intensive learning environments (Mu and Yanchinda, 2024).

To ensure adequate representation, a stratified random sampling technique was employed across multiple universities and disciplines (Nguyen et al., 2021). Stratification was applied by dividing the target population into predefined strata based on (a) university type (public vs. private), (b) academic discipline group (management, engineering, social sciences, health sciences), and (c) year of study (undergraduate vs. postgraduate). Each university provided student enrollment lists broken down by these categories, which collectively served as the sampling frame. Within each stratum, respondents were selected randomly using computer-generated random numbers to ensure proportional representation. This procedure ensured that the final sample reflected the diversity of programs and academic levels within the participating institutions.

Questionnaires were administered in three separate waves over a period of several weeks. In the first wave, 520 questionnaires were distributed and 468 were returned. Of these, 32 were incomplete and therefore removed from the dataset. Wave 2 was administered 3 weeks after Wave 1, using unique matching keys to ensure respondent continuity, yielding 436 valid responses, with 24 removed due to unmatched identifiers. Wave 3 was administered 2 weeks after Wave 2, resulting in 412 returned questionnaires, of which 17 were excluded due to missing values or patterned responses. A final sample of 395 students was retained for analysis. To promote participation, respondents were informed through a detailed cover letter describing the purpose of the study, the voluntary nature of their involvement, and assurances of anonymity and confidentiality.

The demographic characteristics of the sample provided additional insight into the respondents’ profiles. Approximately 54% of the participants were female and 46% were male, reflecting a relatively balanced gender distribution. The majority of respondents were between the ages of 18 and 22 years (62%), representing typical undergraduate cohorts, while 28% fell within the 23 to 26 age range, mainly postgraduate students, and the remaining 10% were above 26 years, indicating the presence of mature or continuing learners. In terms of academic major, 31% of the students were enrolled in management and business-related programs, 27% in engineering and technology, 22% in social sciences and humanities, and 20% in health sciences and allied fields. With regard to year of study, 38% were first- and second-year students, 42% were in their third or fourth year, and 20% were pursuing master’s or doctoral programs. Collectively, these demographic distributions ensured diversity across gender, age, academic field, and level of study, thereby enhancing the representativeness of the final sample of 395 respondents.

## Measures

All constructs were measured using previously validated scales, adapted to the student context. The SECI model of knowledge creation was measured through four dimensions: socialization, externalization, combination, and internalization, each captured with items adapted from prior research (Nonaka et al., 1996). Sample items included “I often share experiences with my peers” (socialization) and “I document ideas into a more concrete form” (externalization). Consistent with recent operationalizations of SECI in educational settings (Avdimiotis et al., 2022; Guzik et al., 2025; Żatuchin, 2024, 2025), each dimension was adapted to reflect student interactions and learning practices.

Absorptive capacity was assessed across three pathways—acquisition, assimilation, and transformation—drawing upon scales from George et al. (2005) and Zahra and George (2002, 2017). Example items included “I acquire necessary knowledge from external sources” (acquisition), “I analyze and interpret acquired knowledge” (assimilation), and “I combine new knowledge with existing knowledge” (transformation). These pathways were structured in line with contemporary applications of multi-dimensional absorptive capacity in innovation-focused research (Hassan et al., 2025; Hassan et al., 2023), ensuring alignment with capability-based knowledge frameworks.

Students’ creative behavior was measured using the scale developed by Tierney and Farmer (2002), with items such as “I often come up with new and practical ideas to improve performance.” All items were rated on a five-point Likert scale ranging from 1 = strongly disagree to 5 = strongly agree. This combination of validated measures provides a robust foundation for capturing the knowledge-creation processes, capability mechanisms, and creative outcomes central to this study.

## Analysis

This study applied partial least squares structural equation modeling (PLS-SEM) using SmartPLS 3.3.3 to examine the proposed relationships. PLS-SEM was chosen because it is particularly appropriate for models that focus on prediction and theory development, involve multiple latent constructs, and incorporate mediation effects. As highlighted by Hair et al. (2017), PLS-SEM is recommended when the aim is to maximize explained variance in endogenous variables rather than to confirm well-established theories. In line with recent guidelines (Hair et al., 2014; Hair et al., 2019), PLS-SEM is also advantageous for handling complex models with both reflective and higher-order constructs such as the SECI model. To test the significance of the structural paths and indirect effects, bootstrapping with 5,000 resamples was employed, which provides robust estimates of standard errors, t-values, and *p*-values.

## Results

The reliability and convergent validity of the constructs were first examined. Table 1 reports item loadings, Cronbach’s alpha (CA), composite reliability (CR), and average variance extracted (AVE). Consistent with the guidelines of Hair et al. (2017), the majority of item loadings exceeded the recommended threshold of 0.70, thereby confirming indicator reliability. Although a few loadings fell slightly below 0.70 (e.g., ACQ1 = 0.596; ASS2 = 0.583; COM3 = 0.549), these were retained because the overall construct reliability and validity indices were well above the required thresholds. Specifically, Cronbach’s alpha values ranged from 0.761 to 0.827, surpassing the 0.70 benchmark, while CR values ranged from 0.843 to 0.880, well above the minimum cut-off of 0.70 (Hair et al., 2014). The AVE values for all constructs were above 0.50, confirming convergent validity (Fornell and Larcker, 1981). Collectively, these results demonstrate that the constructs are measured reliably and exhibit sufficient convergence.

**Table 1:** Reliability and validity tests.

Items	Loadings	CA	CR	AVE
Acquisition		0.707	0.819	0.534
ACQ1	0.596			
ACQ2	0.796			
ACQ3	0.751			
ACQ4	0.764			
Assimilation		0.792	0.852	0.510
ASS1	0.702			
ASS2	0.583			
ASS3	0.742			
ASS4	0.698			
ASS5	0.692			
ASS6	0.777			
Creative behavior		0.719	0.843	0.642
CB1	0.824			
CB2	0.845			
CB3	0.731			
Combination		0.827	0.88	0.599
COM1	0.848			
COM2	0.768			
COM3	0.549			
COM4	0.821			
COM5	0.844			
Externalization		0.752	0.858	0.668
EXT1	0.879			
EXT2	0.776			
EXT3	0.794			
Internalization		0.788	0.864	0.617
INT1	0.643			
INT2	0.843			
INT3	0.87			
INT4	0.767			
Socialization		0.761	0.847	0.585
SOC1	0.76			
SOC2	0.852			
SOC3	0.593			
SOC4	0.829			
Transformation		0.775	0.846	0.525
TRA1	0.71			
TRA2	0.688			
TRA3	0.689			
TRA4	0.74			
TRA5	0.79			

Discriminant validity was assessed using the heterotrait–monotrait ratio (HTMT), as shown in Table 2. Following the criteria suggested by Henseler et al. (2015), HTMT values should be below 0.90 (liberal criterion) or 0.85 (conservative criterion) to establish discriminant validity. In this study, all HTMT ratios were below 0.90, with the highest observed value being 0.885 between assimilation and combination. This indicates that although some constructs, such as assimilation and combination, were closely related conceptually, they remained empirically distinct. These findings affirm that the latent constructs capture unique dimensions of the SECI model, absorptive capacity pathways, and creative behavior without problematic overlap.

**Table 2:** Discriminant validity—HTMT ratio.

Construct	1	2	3	4	5	6	7	8
1. Acquisition								
2. Assimilation	0.622							
3. Combination	0.757	0.885						
4. Creative behavior	0.844	0.867	0.852					
5. Externalization	0.522	0.877	0.482	0.78				
6. Internalization	0.768	0.835	0.768	0.871	0.536			
7. Socialization	0.749	0.638	0.781	0.815	0.439	0.845		
8. Transformation	0.371	0.473	0.46	0.519	0.321	0.451	0.339	

The higher-order construct (HOC) of the SECI model of knowledge creation was also assessed, and the results are presented in Table 3. The SECI model, conceptualized as a second-order factor comprising combination, externalization, internalization, and socialization, demonstrated satisfactory reliability and validity. The Cronbach’s alpha for the HOC was 0.815, exceeding the 0.70 threshold, while the CR was 0.880, above the minimum cut-off of 0.70, thus establishing internal consistency (Hair et al., 2019). The AVE of 0.649 was greater than the recommended value of 0.50, providing evidence of convergent validity. Furthermore, the loadings of the four lower-order dimensions on the SECI construct were high (ranging from 0.659 to 0.861), confirming that these dimensions strongly reflect the higher-order knowledge creation process. Together, these results validate the SECI model as a robust multidimensional construct for subsequent structural testing.

**Table 3:** Construct reliability and validity of HOC.

Dimensions	Loadings	CA	CR	AVE
SECI model of knowledge creation		0.815	0.88	0.649
Combination	0.855			
Externalization	0.659			
Internalization	0.861			
Socialization	0.829			

The results of the structural model are summarized in Table 4. The SECI model of knowledge creation showed a significant direct effect on creative behavior (*β* = 0.598, *p* < 0.001), indicating that the knowledge creation process plays a central role in fostering students’ creative outcomes. Moreover, SECI had significant effects on acquisition (*β* = 0.670, p < 0.001), assimilation (*β* = 0.827, *p* < 0.001), and transformation (*β* = 0.401, *p* < 0.001), confirming its predictive power over all three absorptive capacity pathways. Among these mediators, acquisition (*β* = 0.146, *p* = 0.015) and transformation (*β* = 0.090, *p* = 0.023) exerted significant positive effects on creative behavior, whereas assimilation was non-significant (*β* = 0.066, *p* = 0.308). Mediation analysis further revealed significant indirect effects of SECI on creative behavior through acquisition (*β* = 0.098, *p* = 0.019) and transformation (*β* = 0.036, *p* = 0.047), but not through assimilation (*β* = 0.054, *p* = 0.310). These findings suggest that while acquisition and transformation represent effective pathways through which knowledge creation enhances creativity, assimilation does not act as a meaningful bridge.

**Table 4:** Structural equation model results.

Relationships	*B*	S.E.	*T*	*p*
Direct effects				
SECI model of knowledge creation → Creative behavior	0.598	0.082	7.302	0.000
SECI model of knowledge creation → Acquisition	0.670	0.048	14.004	0.000
SECI model of knowledge creation → Assimilation	0.827	0.017	47.29	0.000
SECI model of knowledge creation → Transformation	0.401	0.061	6.604	0.000
Acquisition → Creative behavior	0.146	0.060	2.452	0.015
Assimilation → Creative behavior	0.066	0.064	1.021	0.308
Transformation → Creative behavior	0.090	0.039	2.285	0.023
Indirect effects				
SECI model of knowledge creation → Acquisition → Creative behavior	0.098	0.042	2.356	0.019
SECI model of knowledge creation → Assimilation → Creative behavior	0.054	0.054	1.017	0.310
SECI model of knowledge creation → Transformation → Creative behavior	0.036	0.018	1.995	0.047

Finally, predictive relevance was assessed through blindfolding analysis, and the results are shown in Table 5. The Stone–Geisser *Q*^2^ values for acquisition (0.234), assimilation (0.322), creative behavior (0.398), and transformation (0.074) were all greater than zero, thereby confirming predictive relevance of the model (Hair et al., 2019). Notably, the highest predictive relevance was observed for creative behavior (*Q*^2^ = 0.398), suggesting that the model has strong predictive accuracy for the main outcome variable. The *Q*^2^ values for acquisition and assimilation were moderate, while transformation exhibited a relatively smaller value (0.074), indicating weaker but still acceptable predictive capacity. Overall, the blindfolding results reinforce the robustness of the structural model by confirming its predictive power across key constructs.

**Table 5:** Blindfolding analysis.

Construct	SSO	SSE	*Q*^2^ (=1-SSE/SSO)
Acquisition	1,580	1209.911	0.234
Assimilation	2,370	1607.52	0.322
Creative behavior	1,185	713.49	0.398
SECI model of knowledge creation	1,580	1,580	
Transformation	1975	1827.874	0.074

**Figure 2:**
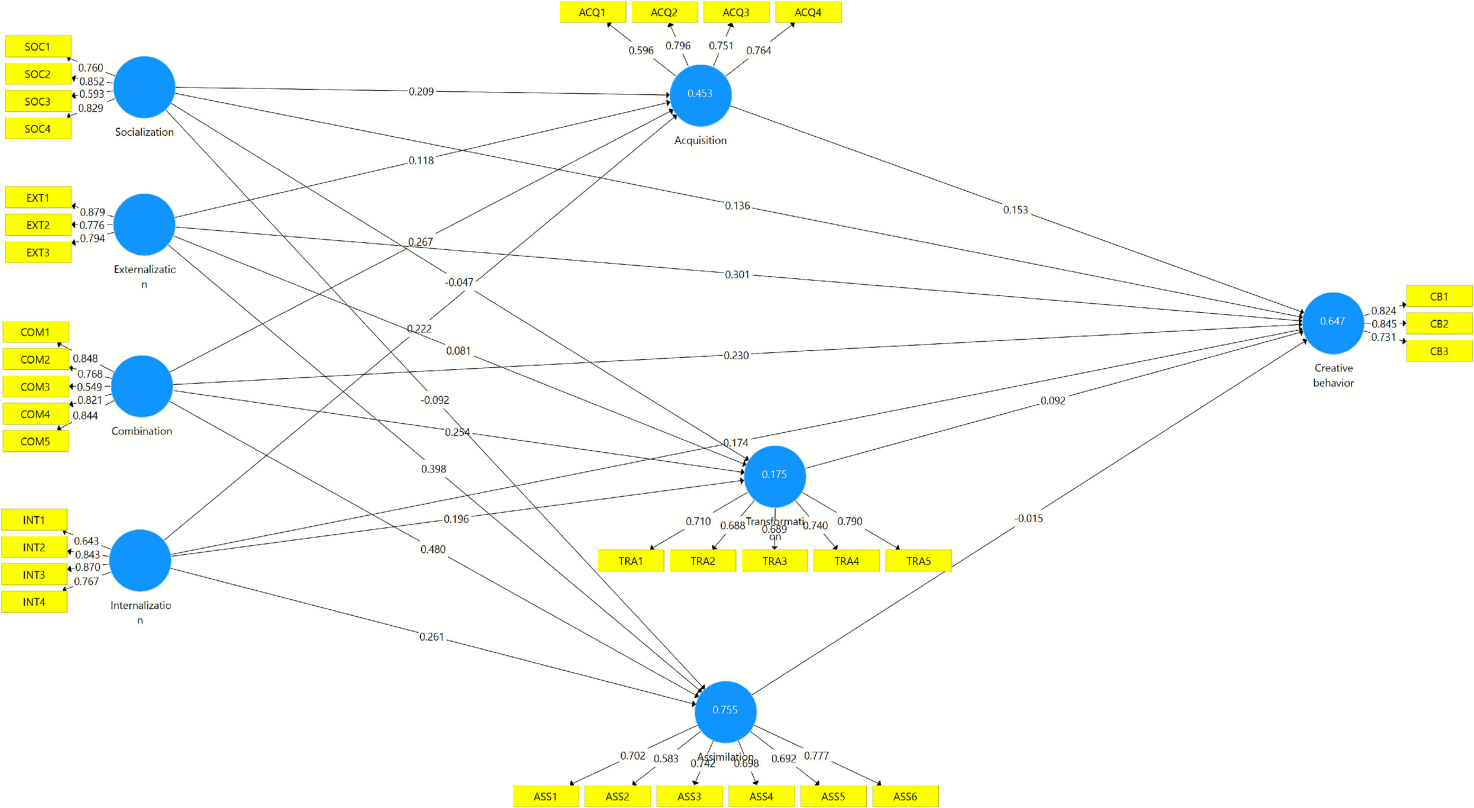
Structural model with lower order constructs.

**Figure 3:**
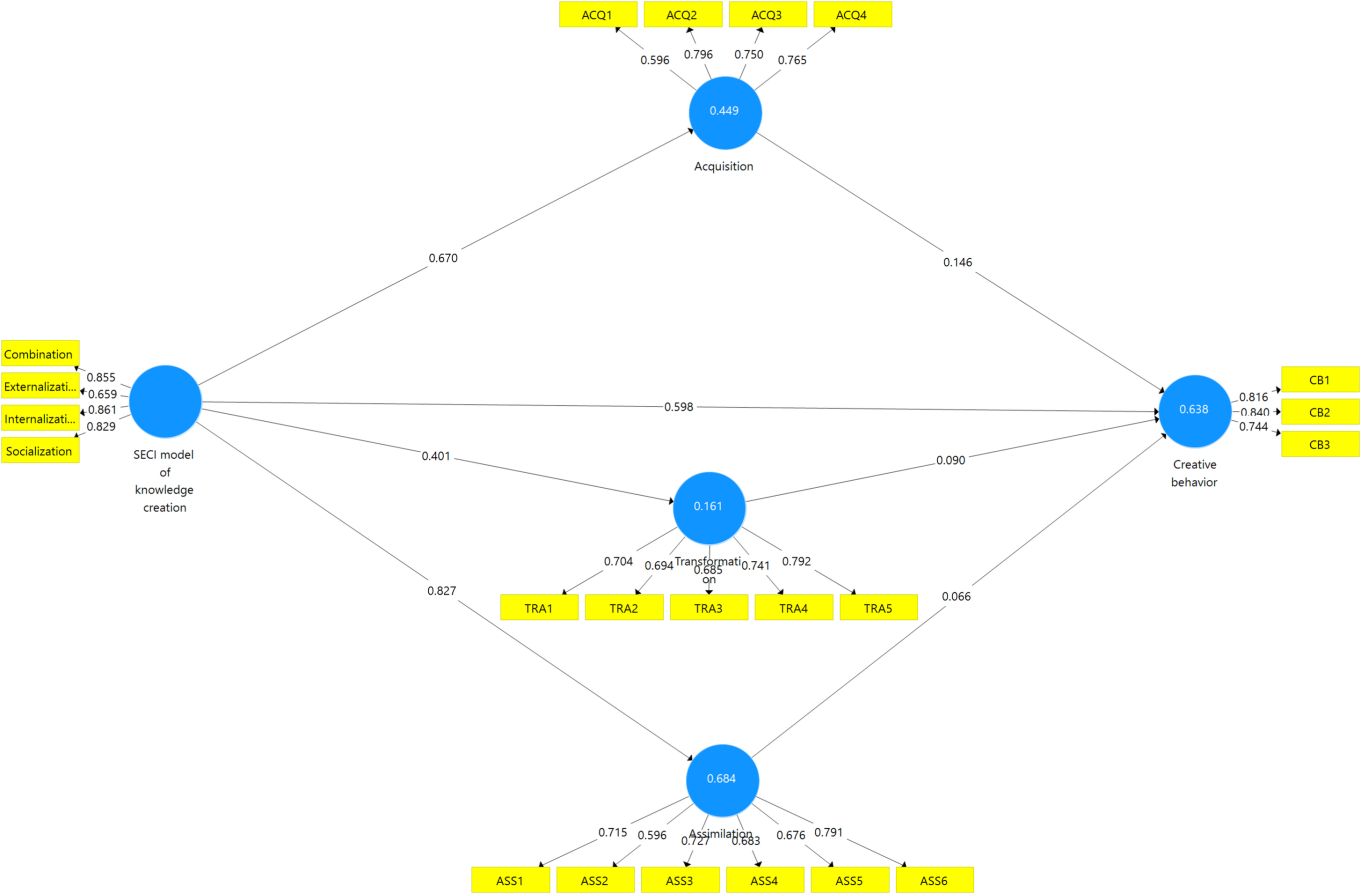
Structural model with higher-order constructs.

In addition, the measurement and structural models are illustrated in Figures 2, 3. Figure 2 presents the structural model with the lower-order constructs, demonstrating the relationships between the SECI model’s four dimensions, the absorptive capacity pathways, and students’ creative behavior. Figure 3 extends this representation by depicting the higher-order construct (HOC) of the SECI model of knowledge creation, where the four dimensions (combination, externalization, internalization, and socialization) are modeled as reflective indicators of a unified second-order construct. These visualizations enhance the clarity of the model by showing both the multidimensional nature of knowledge creation and its predictive links to absorptive capacity and creative outcomes.

## Discussion

The primary aim of this study was to examine how the SECI model of knowledge creation fosters students’ creative behavior both directly and indirectly through absorptive capacity pathways. By applying a time-lagged design and testing both lower-order and higher-order constructs (Hair et al., 2019; Podsakoff et al., 2003), the study provides novel empirical evidence for an integrative framework that, to our knowledge, has not previously been tested in its entirety. The findings reveal that SECI not only exerts a significant direct influence on creative behavior but also strengthens students’ acquisition and transformation capabilities, while its effect on assimilation is more limited. These results advance theory by demonstrating how SECI functions as a unified knowledge-creation capability that activates specific cognitive mechanisms rather than operating as four isolated processes. This clarifies the internal logic of knowledge-creation theory and offers new insights into how SECI interacts with absorptive pathways in educational settings.

The first hypothesis, which predicted a direct positive effect of SECI on creative behavior, was strongly supported. This confirms that the processes of socialization, externalization, combination, and internalization enable students to generate and apply new ideas creatively. Consistent with earlier organizational research showing that SECI drives innovation performance (Flores Torres et al., 2021; Zhang et al., 2025), the present study demonstrates that these mechanisms are equally relevant at the student level. Recent educational studies further reinforce this point, showing that SECI-inspired designs stimulate creativity by converting experiential knowledge into explicit insights (Guzik et al., 2025; Żatuchin, 2024, 2025). Thus, SECI emerges as a foundational knowledge-creation capability that directly nurtures creativity in higher education. Importantly, the current findings show that SECI does more than provide structural opportunities for knowledge sharing—it actively shapes students’ cognitive flexibility, reflective habits, and willingness to experiment. These psychological shifts represent previously overlooked mechanisms through which knowledge-creation processes stimulate creative outcomes (Cheng, 2023), offering a more nuanced account of how SECI contributes to creative capacity-building. Therefore, this study extends SECI theory by demonstrating its role as a personal-level developmental resource rather than merely an organizational knowledge-management tool.

The second hypothesis, predicting that SECI would positively influence acquisition, also received strong empirical support. Students who engaged in SECI processes were more likely to identify, capture, and access new external knowledge. This result aligns with prior findings that collaborative and dialogical learning environments increase learners’ ability to acquire knowledge inputs (Hu et al., 2023; Waena and Hikmawati, 2022). It also extends absorptive capacity theory (Zahra and George, 2002) by showing that SECI processes, traditionally studied in organizational contexts, provide students with enhanced acquisition skills essential for creativity. More specifically, the current findings indicate that socialization and externalization act as cognitive triggers that increase curiosity, information scanning, and the motivation to seek diverse knowledge sources, consistent with recent arguments by Lenart-Gansiniec et al. (2022). This positions SECI as a key antecedent of potential absorptive capacity, offering theoretical clarity on how knowledge-creation practices stimulate students’ knowledge-seeking behaviors.

The third hypothesis posited that SECI would positively predict transformation, and the results confirmed this relationship. Transformation reflects the recombination of new and existing knowledge, and the findings suggest that SECI creates the conditions for such reconfiguration. This aligns with prior work emphasizing that transformation is a key step in generating novelty from accumulated knowledge (Wang and Zhang, 2024; Żatuchin, 2025). Recent educational evidence similarly shows that transformational learning processes encourage innovation in problem-solving (Żatuchin, 2024). By embedding transformation within the SECI framework, the current study extends knowledge creation theory to the student context, highlighting that knowledge recombination is a critical enabler of creativity. The findings also provide empirical insight into how combination and externalization jointly support transformational learning by facilitating knowledge restructuring and cross-contextual application (Guzik et al., 2025). This contributes theoretically by integrating realized absorptive capacity with SECI activity, strengthening conceptual bridges between these two models.

The fourth hypothesis, predicting that SECI would positively influence assimilation, was also supported. The results indicate that SECI enables students to interpret and internalize knowledge more effectively. This finding is consistent with studies suggesting that structured knowledge activities improve sense-making (Asbari and Asbari, 2025; Barreto and Abarca, 2025; Zhang et al., 2025). Assimilation thus plays a key role in helping students cognitively organize new inputs. However, as the later mediation analysis indicates, assimilation alone does not directly translate into creativity, which distinguishes its role from acquisition and transformation. This result supports theoretical claims that potential absorptive capacity alone is insufficient to produce innovative outcomes without progression into realized capacity (Li et al., 2018; Lo and Tian, 2020). The present findings therefore clarify that comprehension must be complemented by reconfiguration (transformation) for creativity to emerge, refining assumptions embedded in both absorptive capacity and creativity research.

The fifth hypothesis examined whether acquisition mediated the SECI–creativity link. The results supported this mediation, establishing acquisition as a crucial pathway. This finding confirms that the ability to acquire external knowledge is a precursor to creativity (Gilson, 2024) and resonates with recent evidence showing that acquisition enables students to transform learning experiences into innovative outputs (Fischer-Schöneborn et al., 2025). This pathway underscores that creativity in higher education is seeded in knowledge-seeking behavior facilitated by SECI. Importantly, the mediation effect demonstrates that SECI’s influence on creativity is amplified when learners engage in proactive information gathering, supporting the argument that informational breadth enhances cognitive flexibility (Wan et al., 2021). This strengthens theoretical clarity on why acquisition consistently emerges as the most influential absorptive pathway in creativity-focused models.

The sixth hypothesis tested transformation as a mediator, and the results again provided support, though with a smaller effect than acquisition. This suggests that while transformation contributes to creativity, its role is complementary rather than dominant. Consistent with Wang and Zhang (2024), transformation is the mechanism through which knowledge is reconfigured, and our results extend this line of reasoning into education by showing that transformational activities—such as applying knowledge to new contexts—support students’ creative outcomes. The findings also support the notion that creativity results from iterative cycles of knowledge integration and reapplication rather than from single exposures to information (Maras et al., 2024). This provides theoretical refinement by confirming that transformation acts as a secondary but necessary pathway linking SECI to creative performance.

The seventh hypothesis predicted that assimilation would mediate the SECI–creativity relationship. Contrary to expectations, this pathway was not significant. Although SECI strongly predicts assimilation, the ability to interpret and understand knowledge alone does not directly drive creative behavior. This finding echoes prior arguments that potential absorptive capacity (acquisition and assimilation) must be converted into realized capacity (transformation and exploitation) to yield innovation (Flores Torres et al., 2021; Li et al., 2018; Lo and Tian, 2020). In educational contexts, this highlights that comprehension, while foundational, does not guarantee creativity unless coupled with processes that involve reconfiguration and application. This non-significant mediation enriches theory by clarifying that understanding is a necessary but insufficient condition for creativity, correcting assumptions that deeper comprehension inherently leads to innovation. Such insights contribute to a more accurate representation of absorptive capacity dynamics within creative learning processes.

Moreover, a notable methodological contribution lies in the validation of SECI as a higher-order construct. Modeling SECI both as separate dimensions and as a unified second-order factor demonstrates its parsimony and predictive utility (Hair et al., 2019). The findings confirm that SECI’s four processes cohere into a single overarching capability that significantly shapes absorptive capacity and creativity. This dual modeling enriches theory by showing that knowledge creation can be conceptualized both dimensionally and holistically, providing flexibility for future researchers. The adoption of reflective–reflective higher-order modeling using PLS-SEM also demonstrates methodological rigor and reinforces its suitability for complex, multistage theoretical models in education research (Chin, 2010). This methodological insight offers a template for future scholars seeking to operationalize multi-dimensional knowledge constructs in predictive frameworks.

In addition to these contributions, this study incorporates several methodological strengths. The use of PLS-SEM is particularly appropriate given the predictive focus and the complexity of the model, which includes both mediating mechanisms and a higher-order construct. The application of the SECI knowledge-creation model in a student context also represents a theoretical strength, as it provides a robust and well-established framework for examining how knowledge processes translate into creative behavior. Together, these strengths enhance the rigor, clarity, and interpretive value of the findings.

Although SECI significantly predicted assimilation, the non-significant mediating effect suggests that comprehension alone may not translate into creative behaviors without deeper cognitive activation. One possible explanation is cognitive overload, where students exposed to dense or complex knowledge may focus on understanding rather than generating new ideas, thereby limiting the transition from assimilation to creative output. Another plausible explanation relates to passive or surface learning styles, which emphasize information absorption over application; students who rely heavily on memorization may assimilate knowledge effectively but fail to reconfigure it creatively. Prior research similarly reports that interpretive learning does not automatically trigger creativity unless accompanied by reflective or higher-order processing [e.g., contrasting findings in Zhang et al. (2025) and Mohamed (2021)]. Furthermore, studies in educational psychology indicate that assimilation contributes more to stability and comprehension than to novelty generation, whereas transformation processes better predict innovative outcomes. These contrasting insights highlight that assimilation may serve as a foundational but insufficient precursor to creativity, requiring additional cognitive mechanisms to activate its creative potential.

### Practical implications

The findings of this study carry several important implications for higher education practice and policy. First, the results suggest that universities should design learning environments that foster systematic knowledge creation processes aligned with the SECI model. Encouraging activities such as peer sharing (socialization), structured articulation of ideas (externalization), collaborative problem-solving (combination), and reflection on experiences (internalization) can enhance students’ absorptive capacity and prepare them for creative performance. Instructors can embed these practices into coursework through team projects, reflective writing, and interactive workshops that simulate real-world knowledge creation contexts. For example, curriculum designers may incorporate SECI-driven learning cycles by structuring weekly modules that alternate between experiential tasks (socialization), concept articulation assignments (externalization), integrated group analysis (combination), and reflective journals (internalization), thereby ensuring that students repeatedly engage in all stages of knowledge conversion.

Second, the evidence that acquisition and transformation significantly mediate the SECI–creativity link highlights the need for educators to provide opportunities for students to both acquire new knowledge from diverse external sources and to reconfigure that knowledge in novel ways. This can be operationalized through experiential learning programs, partnerships with industry, and exposure to interdisciplinary coursework. Such practices strengthen students’ abilities not only to gather information but also to adapt and apply it creatively, thereby equipping them with innovation-oriented skills valued in modern workplaces. In this regard, universities can introduce interdisciplinary innovation studios where students from different majors collaboratively reframe real organizational problems and develop creative prototypes, directly activating both acquisition and transformation pathways.

Third, the non-significant role of assimilation implies that mere comprehension and interpretation of knowledge is insufficient to foster creativity. While understanding is foundational, institutions must ensure that curricula extend beyond memorization or rote learning and instead promote application and recontextualization. This calls for a pedagogical shift toward active learning, case-based teaching, and project-based assessments that encourage students to move beyond assimilation toward transformation and creation. This shift ensures that students actively practice applying knowledge in varied contexts, which accelerates deep learning and strengthens their capacity to produce original solutions.

Finally, the validation of SECI as a higher-order construct reinforces the value of approaching knowledge creation holistically in educational settings. Rather than treating socialization, externalization, combination, and internalization as isolated activities, universities should recognize their synergistic power in building student creativity. Institutional policies should therefore encourage integrated approaches—such as innovation labs, interdisciplinary seminars, and knowledge-sharing platforms—that combine the four dimensions into a coherent capability. By doing so, higher education institutions not only cultivate creativity among students but also strengthen their own roles as engines of innovation in society. When implemented effectively, these integrated approaches give students continuous, structured exposure to creative thinking processes, helping them develop resilience, perspective-taking, and the ability to innovate collaboratively.

## Limitations

Although this study makes several theoretical and practical contributions, certain limitations should be acknowledged. First, the data were collected from university students in China, which may limit the generalizability of the findings to other cultural and educational contexts. Cultural norms influence how knowledge is shared, interpreted, and transformed (Hofstede, 2001), and future research should examine whether the integrative SECI–absorptive capacity–creativity framework operates similarly in different cultural or institutional environments, such as Western universities or cross-cultural student populations. Given this contextual boundary, future work should also consider replicating the model across diverse educational systems to strengthen generalizability.

Second, the study employed a self-reported survey design, which, despite the use of a three-wave time-lagged method to reduce common method variance (Podsakoff et al., 2003), remains subject to biases such as social desirability and self-perception. Future studies could benefit from incorporating multiple data sources, including peer evaluations, instructor assessments, or objective measures of creativity (e.g., project evaluations, innovation competitions), to triangulate results and enhance validity.

Third, while the current study examined acquisition, assimilation, and transformation as key absorptive capacity pathways, it did not include exploitation, which is also emphasized in absorptive capacity theory (Zahra and George, 2002). Future research should expand the model by incorporating exploitation to capture the full spectrum of absorptive capacity, thereby offering a more comprehensive understanding of how knowledge creation translates into creative outcomes.

Fourth, the model was tested using partial least squares structural equation modeling (PLS-SEM), which is appropriate for prediction-oriented models (Hair et al., 2017). However, future work could apply alternative methods such as covariance-based SEM to examine model fit in confirmatory contexts, or use longitudinal and experimental designs to assess causality more robustly.

Finally, the non-significant role of assimilation in predicting creative behavior, although theoretically meaningful, warrants deeper investigation. Future research could explore boundary conditions—such as learning styles, digital learning environments, or team-based interactions—that may moderate the role of assimilation. Such extensions could refine understanding of when assimilation contributes to creativity and when it remains a background enabler rather than a direct predictor.

In addition, future studies could incorporate other influential variables—such as learning style and teamwork—as potential moderators, since many individual and contextual factors may shape how students acquire, interpret, and apply knowledge creatively. Including these moderators would provide a richer understanding of the mechanisms through which SECI processes influence creativity.

## Conclusion

This study set out to explore how the SECI model of knowledge creation fosters students’ creative behavior through the absorptive capacity pathways of acquisition, assimilation, and transformation. By adopting a time-lagged design and testing both lower-order and higher-order models, the research offers fresh empirical evidence for a framework that has not previously been examined in its entirety. The findings demonstrate that acquisition and transformation act as significant pathways linking knowledge creation to creativity, while assimilation, although positively influenced by SECI, does not directly contribute to creative outcomes. This nuance enriches absorptive capacity theory by clarifying which dimensions drive creativity and which function more as supportive foundations.

The study contributes to theory by validating the SECI model as a higher-order construct in educational contexts, bridging organizational knowledge creation and absorptive capacity research, and extending their application to the student level. It also provides practical guidance for educators and institutions, highlighting the need to emphasize not only knowledge comprehension but also the acquisition and transformation of knowledge to stimulate creative behaviors. Although conducted within a single cultural and educational context, the study highlights the potential of integrated knowledge creation and absorptive capacity frameworks to inform pedagogy and curriculum design worldwide.

## Data Availability

The raw data supporting the conclusions of this article will be made available by the authors, without undue reservation.
